# Supplementary material for: Unconventional Hall response in the quantum limit of HfTe5
Source: Nat Commun. 2020 Nov 23;11:5926. doi: 10.1038/s41467-020-19773-y (PMC7683529; doi:10.1038/s41467-020-19773-y)
Supplement: Supplementary file 1 — Supplementary Information [file 41467_2020_19773_MOESM1_ESM.pdf]

Supplementary Information for the manuscript

Unconventional Hall response in the quantum limit of $HfTe_5$

S. Galeski[1], X. Zhao[2,3,4], R. Wawrzyńczak[1], T. Meng[5], T. Förster[6], P. M. Lozano[7], S. Honnali[1], N. Lamba[1], T. Ehmcke[5], A. Markou[1], Q. Li.[7], G. Gu[7], W. Zhu[2,4], J. Wosnitza[6,8], C. Felser[1], G. F. Chen[2,3,4], J. Gooth[1,8]

[1]*Max Planck Institute for Chemical Physics of Solids, Nöthnitzer Straße 40, 01187 Dresden, Germany.*

[2]*Institute of Physics and Beijing National Laboratory for Condensed Matter Physics, Chinese Academy of Sciences, Beijing 100190, China.*

[3]*Songshan Lake Materials Laboratory, Dongguan, Guangdong 523808, China.*

[4]*School of Physics Science, University of Chinese Academy of Sciences, Beijing 100049, China.*

[5]*Institute of Theoretical Physics, Technische Universität Dresden, 01062 Dresden and Würzburg-Dresden Cluster of Excellence ct.qmat, Technische Universität Dresden, 01062 Dresden, Germany.*

[6]*Hochfeld-Magnetlabor Dresden (HLD-EMFL) and Würzburg-Dresden Cluster of Excellence ct.qmat, Helmholtz-Zentrum Dresden-Rossendorf, 01328 Dresden, Germany.*

[7]*Condensed Matter Physics and Materials Science Department, Brookhaven National Laboratory, Upton, NY, USA.*

[8]*Institut für Festkörper- und Materialphysik, Technische Universität Dresden, 01062 Dresden, Germany.*

Table of Content

Supplementary note 1: Charge-carrier density and mobility from Hall measurements

Supplementary note 2: Mapping of the Fermi surface by analyzing Shubnikov-de Haas oscillations

Supplementary note 3: Comment on the calculation of the Hall conductivity tensor element

Supplementary note 4: Theory of the three-dimensional Hall effect

Supplementary Fig. S1 – S17

Supplementary Table S1

Supplementary References

Supplementary note 1: Charge-carrier density and mobility from Hall measurements

From linear fits of the low-field Hall measurements (Supplementary Fig. S2a), we obtain the temperature-dependent dominant charge-carrier concentration $n = (\mathrm{d}\rho_{xy}/\mathrm{d}|B|\cdot e)^{-1}$ (Supplementary Fig. S2b) and the average mobility $\mu = (\rho_{xx,0}\, en)^{-1}$ (Supplementary Fig. S2c) of $HfTe_5$, using a single-band model.

Supplementary note 2: Mapping of the Fermi surface by analyzing Shubnikov-de Haas oscillations

We mapped the Fermi surface of our $HfTe_5$ samples by analyzing Shubnikov-de Haas oscillations in the temperature (T)-dependent longitudinal magneto-electrical resistivity $\rho_{xx}(\boldsymbol{B})$.[1] In these measurements, the electrical current is applied along the x-axis with the magnetic field set along the x, y, and z directions. The results of our analysis are summarized in Table S1. For all directions, we observe single frequencies $B_{F,i}$, as shown in Fig. 1, Supplementary Fig. S4, and Supplementary Fig. S5, with i being the direction of applied magnetic field. The Landau index N is related to the Fermi surface as $2\pi(N+g) = B_{F,i}/\boldsymbol{B}$, where the phase shift g is approximately zero for all samples. From the slope of linear fits in Landau-index fan diagrams, we extract $B_{F,i}$ and use the Onsager relation $B_{F,i} = (\hbar/2\pi e)S_{F,i}$ to extract the Fermi surface cross-section $S_{F,i}$. Here, $\hbar$ is the reduced Planck constant and e is the electron charge. Under the assumption of an ellipsoidal Fermi surface, the Fermi wave vectors are then

given by $k_{F,x} = \sqrt{S_{F,y}S_{F,z}}/\sqrt{\pi S_{F,x}}$, $k_{F,y} = \sqrt{S_{F,x}S_{F,z}}/\sqrt{\pi S_{F,y}}$ and $k_{F,z} = \sqrt{S_{F,x}S_{F,y}}/\sqrt{\pi S_{F,z}}$. The $k_{F,i}$ relate then directly to the Fermi wave length $\lambda_{F,i} = 2\pi/k_{F,i}$.

The resistance amplitude of the maxima in the Shubnikov-de Haas oscillations in $\Delta\rho_{xx}(\boldsymbol{B})$ is proportional to $\chi(\boldsymbol{B})/\sinh[\chi(\boldsymbol{B})]\cdot\exp(\omega_c\tau/\boldsymbol{B})$ with the cyclotron frequency $\omega_c = \frac{e\,|\boldsymbol{B}|}{m_c}$ and $\chi(\boldsymbol{B}) = \frac{2\pi^2 k_B T m_c}{\hbar e|\boldsymbol{B}|}$, where m_c is the cyclotron mass. Hence, when plotting $\sinh[\chi(\boldsymbol{B})]/\chi(\boldsymbol{B})$ against $1/\boldsymbol{B}$, the carrier lifetime τ can be extracted from the slope of the logarithmic (Supplementary Fig. S10c-e). The corresponding effective mass can be extracted from fitting the T-dependence to $\sinh[\chi(\boldsymbol{B})]/\chi(\boldsymbol{B})$ (Supplementary Fig. S10f-h). Assuming that the massive Dirac band exhibits a linear dispersion at low energies, we finally can obtain the effective masses m^* from the cyclotron masses in the x, y and z direction: $m_{c,x} = \sqrt{m_y^* m_z^*}$, $m_{c,y} = \sqrt{m_x^* m_z^*}$ and $m_{c,z} = \sqrt{m_y^* m_x^*}$, respectively. The Fermi velocities $v_{F,i}$ can be further obtained with $v_{F,i}\, m^*_i = \hbar\, k_{F,i}$. Eventually, the average Fermi energy can be estimated using $E_F = (v_{F,x}^2\, \hbar^2 k_{F,x}^2 + v_{F,y}^2\, \hbar^2 k_{F,y}^2 + v_{F,z}^2\, \hbar^2 k_{F,z}^2)^{0.5}$. For sample A we obtain $E_F = (9 \pm 2)$ meV, where the deviation is obtained from the error of the fits in $k_{F,i}$ and $v_{F,i}$.

Supplementary note 3: Comment on the calculation of the Hall conductivity tensor element

We calculate the Hall conductivity tensor element σ_{xy} using $\sigma_{xy} = \rho_{xy}/(\rho_{xx}^2+\rho_{xy}^2)$, assuming that $\rho_{xx} = \rho_{yy}$. However, in general $\sigma_{xy} = \rho_{xy}/(\rho_{xx}\,\rho_{yy} + \rho_{xy}^2)$ with a magnetic field in z-direction. Due to the geometry of the $HfTe_5$ crystals (elongated needles) and its mechanical fragility, performing reliable measurements of ρ_{yy} on our samples is not possible. Instead, we estimate the error of the σ_{xy} using the ratio of Drude resistivities $\rho_{yy}/\rho_{xx} = (n_{xy}e^2\tau_x/\boldsymbol{m_x^*})/\ (n_{xy}e^2\tau_y/\boldsymbol{m_y^*})$ estimated from the quantum lifetimes and effective masses obtained from Shubnikov-de Haas

oscillations on sample A, given in Supplementary Table S1. n_{xy} is the charge-carrier concentration in the *x*-*y*-plane. Based on this analysis we find $\rho_{yy}/\rho_{xx} \approx 0.4$, which results in an error of 2 % in the estimated σ_{xy} at the 3/5 plateau, owing to $\rho_{xx}(\boldsymbol{B}) < \rho_{xy}(\boldsymbol{B})$. Both these errors lay well within the estimated error of $k_{F,z}$ of 10 %. Therefore, the Hall plateaus in ρ_{xy} are expected to be observable in the Hall plateaus in σ_{xy}.

Supplementary note 4: Theory of the three-dimensional Hall effect

Hall effect stacks in momentum space

Two-dimensional electrons subject to a magnetic field $\boldsymbol{B}$ form Landau levels of energy $E_N = (N - \frac{1}{2})\hbar\omega_c$, where $N \geq 1$ is the Landau level index. A three-dimensional Hall system can be understood as stacking of such two-dimensional Hall layers on top of each other. Any finite hopping along the third direction - here taken to be the direction of the magnetic field: the *z*-direction - results in an additional dispersion with the momentum p_z along *z*. The Landau levels consequently form bands of energy $E_N(p_z) = (N - \frac{1}{2})\hbar\omega_c + \frac{{p_z}^2}{2m_z^*}$, where m_z^* is the mass associated with the *z*-dispersion, and it is more convenient to think about a 3D Hall system in momentum space. If the energy $E_N(p_z)$ of a given band N is below the Fermi level E_F for a certain momentum range $\Delta p_{z,N}$ (see Supplementary Fig. S4), then the corresponding 2D-momentum layers (layers of p_x and p_y) are occupied and gapped. Their gap $\Delta_{N'}(p_z)$ is the energy difference of these states to the Fermi level, $\Delta_{N'}(p_z) = E_F - E_N(p_z)$. Each of the occupied momentum-space layers carries one quantum of Hall conductance.

As shown in Supplementary Fig. S5, the density of states of a 3D Hall system is that of a macroscopically degenerate sets of one-dimensional bands: van-Hove singularities at the band bottoms are followed by tails extending to high energies. The degeneracy of these bands equals

the macroscopic degeneracy $J_{LL} = \frac{e\,|\boldsymbol{B}|}{2\,\pi\hbar}\,L_x\,L_y$ of individual Landau levels, where L_i is the length of the system along the i-direction. The total density of states of a 3D Hall system without additional gaps is thus generally finite at the Fermi level, reflecting the fact that the Fermi level intersects Landau level bands.

In an ideal model system, each occupied 2D-momentum layer contributes one quantum of Hall conductance $\frac{e^2}{h}$, giving rise to a total 3D Hall conductivity of $\sigma_{xy} = \sum_N \frac{\Delta p_{z,N}}{2\pi\hbar}\frac{e^2}{h}$. This does, however, not mean that the 3D Hall conductivity is quantized in real samples. First, given that there are gapless bulk states at the Fermi level, disorder-induced couplings of the edge states across the sample can destroy the quantization of the Hall response. Secondly, any small change of the E_F changes $\Delta p_{z,N}$, and thus also σ_{xy}, in some non-universal way. The gapless bulk states also give rise to a finite, non-universal longitudinal conductivity. A truly quantized Hall response can only arise when an additional gap opens at the Fermi level.[2] Consider the example that the chemical potential resides in the lowest Landau level. If the system then forms a density wave with wave vector $G_z = \Delta p_{z,0}/\hbar$, the spectrum is fully gapped, the Hall conductivity is quantized to $\sigma_{xy} = \frac{G_z}{2\pi}\frac{e^2}{h}$, and the longitudinal conductivity vanishes.

Fixed chemical potential vs. fixed particle number

The electric response of a Hall sample can take qualitatively different forms depending on whether an experiment is performed at fixed particle number or at fixed chemical potential, *i.e.* Fermi level.

(i) Fixed chemical potential

If a small sample is strongly connected to large reservoirs, these fix the chemical potential in the sample. Given that the Landau level bands move with magnetic field and because their degeneracy increases linearly with field, a fixed chemical potential implies a varying total electron density n_{tot}. Whenever a Landau-level band bottom crosses the chemical potential, its van Hove singularity induces a kink in the density of electrons in the sample. The Hall response likewise features kinks when a Landau level crosses the chemical potential. These kinks are followed by a plateau-like feature. The longitudinal resistivity depends on the bulk density of states at the Fermi level, and therefore exhibits spikes whenever a Landau-level band bottom crosses E_F. To illustrate this behavior, we extend the treatment of the 2D quantum Hall effect presented in Ref. [3] to three dimensions by including a dispersion of the Landau-level bands and summing over all p_z. Following Ref. [3], we keep track of a Lorentzian level broadening, and also introduce a transport lifetime τ_M (in contrast to the quantum lifetime encoding how long it takes an electron to be scattered into any other state, the transport lifetime encodes that forward scattering does not alter transport). As Ref. [3], we choose the transport lifetime to be ten times larger than the quantum lifetime, which in turn is defined by the inverse level broadening. The resulting electron density, Hall conductivity, and longitudinal resistivity are shown in Supplementary Fig. S6a-c. The quantum limit is reached when the $N = 2$-Landau level band has shifted above the chemical potential such that only states in the $N = 1$-Landau-level band are occupied. In Supplementary Fig. S6a-c, the quantum limit approximately corresponds to 3 T < $|\boldsymbol{B}|$ < 9 T. Above 9 T, also the zeroth Landau-level shifts above the chemical potential (its low-energy tail results in a small residual electron density that disappears when $|\boldsymbol{B}|$ increases further).

(ii) Fixed particle number

In samples containing many electrons, a change of the electron density costs a large charging energy.[4] If such a large sample is only weakly coupled to leads, the particle number rather than the chemical potential is kept fixed when the magnetic field is varied. This in turn requires the chemical potential to vary as a function of magnetic field. Each time a Landau-level bottom crosses the varying chemical potential, the chemical potential exhibits a peak. Using that the Landau-level degeneracy is given by J_{LL}, one furthermore finds that the Hall conductivity is given by $\sigma_{xy} = \frac{e^2}{h}\frac{2\pi\,\hbar}{e\,B}\,n_{tot}$: The Hall conductivity exhibits a featureless $1/|\boldsymbol{B}|$-behaviour. In contrast, the van Hove singularities associated with Landau-level band bottoms still induce spikes in the longitudinal conductivity. This behaviour of a 3D Hall sample at fixed particle number is illustrated in Supplementary Fig. S6d-f.

(iii) Role of level broadening

In a system with fixed particle number, the inclusion of level broadening smoothens out the dependence of the chemical potential on the magnetic field. As a result, the Hall response is slightly closer to the Hall response of a system with fixed chemical potential: we find that the Hall conductivity exhibits small kinks once level broadening is considered.

(iv) Specifics of our samples

In general, a real sample will be in between the two extreme cases of fixed conduction electron (charge carrier) number and fixed chemical potential (Fermi level). This is also apparent from the behavior our samples. At small fields, the Hall response shows slightly smoothened kinks, followed by plateau-like features. This behavior is similar

to the theoretical expectations for a system at fixed chemical potential. We note that our samples are not very large, and that the Dirac pocket is comparably small. Furthermore, at magnetic fields below the quantum limit, the variation of particle density in our toy model at exactly fixed chemical potential is at most about 20%. Finally, the chemical potential does not have to be perfectly conserved for the Hall data to exhibit kinks and plateau-like features: an ideal $1/|\boldsymbol{B}|$-behavior arises only if the chemical potential adjusts perfectly, and if the level broadening is small. Our data, therefore, suggests the presence of localized states that can soak up/release some amount of conduction electrons in order to keep the chemical potential of the sample closer to the chemical potential in the leads. At fields beyond the quantum limit and temperatures at which the $\nu = 3/5$-plateau has not yet developed; the Hall conductivity instead follows a $1/|\boldsymbol{B}|$-behavior. We interpret this behavior as the system's tendency to avoid large changes of the conduction electron density. Finally, the electrons in our samples form a (gapped) Dirac semimetal rather than a quadratic dispersion. As a consequence, the spectrum is particle-hole symmetric. If the Zeemann effect is negligible, all Landau levels except for the zeroth Landau levels above and below the node are spin-degenerate (the zeroth Landau levels are non-degenerate). The levels with $|N| > 1$ shift in field, while the lowest Landau levels are unaffected by the field. With Zeeman splitting, the $|N| > 1$ Landau levels are Zeeman-split and the $N = 1$-Landau levels shift in field. We find that the main difference between a Dirac semimetal and 3D electrons with a quadratic dispersion is the precise location of the various plateaus, but that the main features are qualitatively identical. Most importantly, because the zeroth Landau levels of a Dirac semimetal shift only very weakly at small fields, even when the Zeeman is effect included, the range of occupied momenta in the $N = 1$-band is $\Delta p_{z,1}(B) \approx \Delta p_{z,1}(B = 0) = 2\hbar k_{F,z}$. As a result, the Hall conductivity just above the quantum limit is given by $\sigma_{xy} = \frac{\Delta p_{z,1}}{2\pi\hbar}\frac{e^2}{h} \approx \frac{2k_{F,z}}{2\pi}\frac{e^2}{h}$.

Only at larger fields, when the chemical potential is renormalized substantially, the Hall conductivity drop.

In contrast to 2D quantum Hall systems, the interplay of the different temperature-dependencies of the finite values of ρ_{xx} at its minima and the metal-insulator transition at high fields in $HfTe_5$, cause the position of the minima in $\rho_{xx}(\boldsymbol{B})$ to shift in temperature (Fig 3f). However, importantly, we note that below 800 mK, the positions of the minima up to 5 T remain at fixed positions in magnetic field for all temperatures. This enables the assignment of the magnitude of the plateau-like features in the Hall measurements to the minima of $\rho_{xx}(\boldsymbol{B})$ up to that field in this low-temperature range.

Supplementary Figures

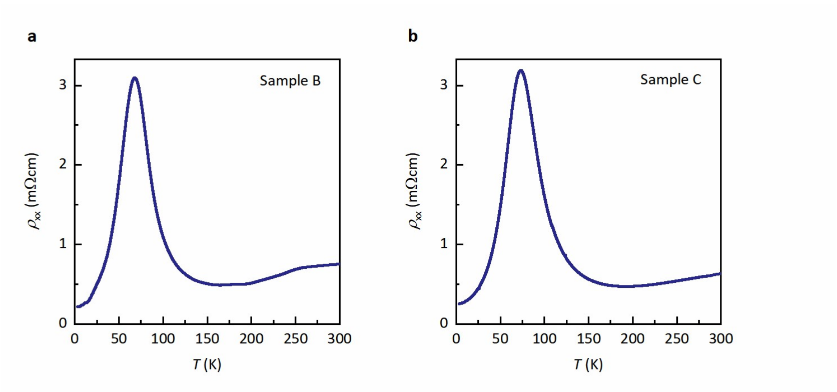

Supplementary Fig. S1. Longitudinal resistivity ρ_{xx} of $HfTe_5$ sample B and C as a function of temperature T at zero magnetic field. a, ρ_{xx} of sample B and **b**, ρ_{xx} of sample C.

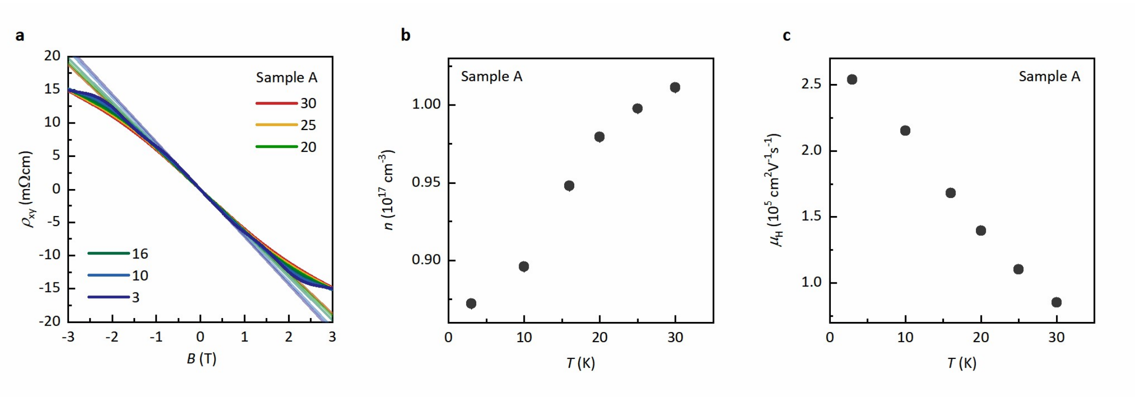

Supplementary Fig. S2. Charge-carrier concentration and Hall mobility of sample A. a, Linear fits of the Hall resistivity at low magnetic fields (fit range ±0.5 T). **b**, Extracted charge-carrier concentration $n = (\mathrm{d}\rho_{xy}/\mathrm{d}|\boldsymbol{B}|\cdot e)^{-1}$ from the slope of the linear fits $\mathrm{d}\rho_{xy}/\mathrm{d}|\boldsymbol{B}|$. Here, e is the electron charge. **c**, Calculated Hall mobility $\mu_H = (\rho_{xx}\cdot e\cdot n)^{-1}$.

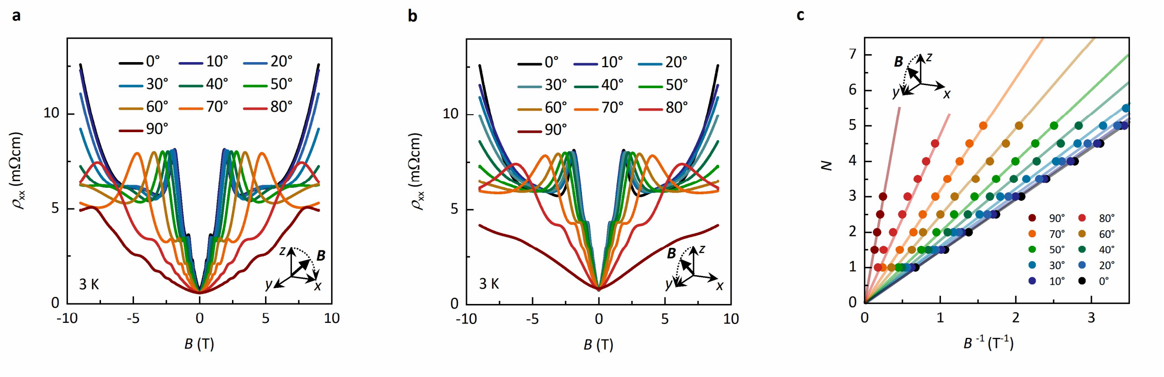

Supplementary Fig. S3. Rotation angle-dependence of the Shubnikov-de Haas oscillations. **a**, ρ_{xx} versus $\boldsymbol{B}$ for various rotation angles of $\boldsymbol{B}$ in the *z*-*x* plane and **b**, ρ_{xx} in the *y*-*x* plane at 3 K. **c,** Landau-level fan diagram for various rotation angles of $\boldsymbol{B}$ in the *y*-*x* plane at 3 K. The data is obtained from the minima of ρ_{xx} in B.

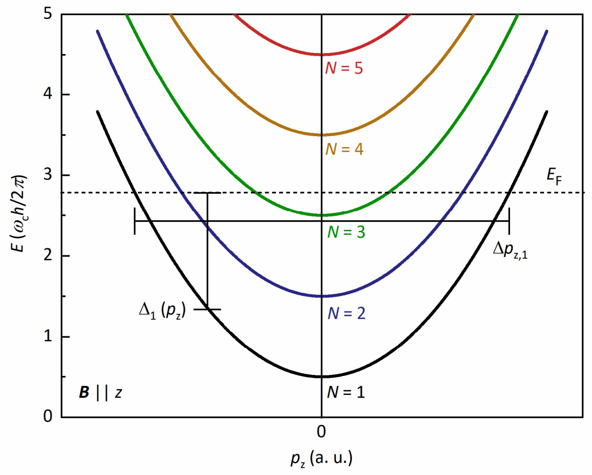

Supplementary Fig. S4. Landau-level band structure of a 3D Hall system. Energy E as a function of momentum p_z for a magnetic field $\boldsymbol{B}$ applied in in z-direction. The Landau levels are indexed as indices N. E_F denotes the Fermi level. $\Delta p_{z,1}$ is the distance between the momentum points of the $N = 1$ Landau level at E_F. $\Delta_1(p_z)$ is the energy difference of the states of the $N = 1$ Landau level and E_F.

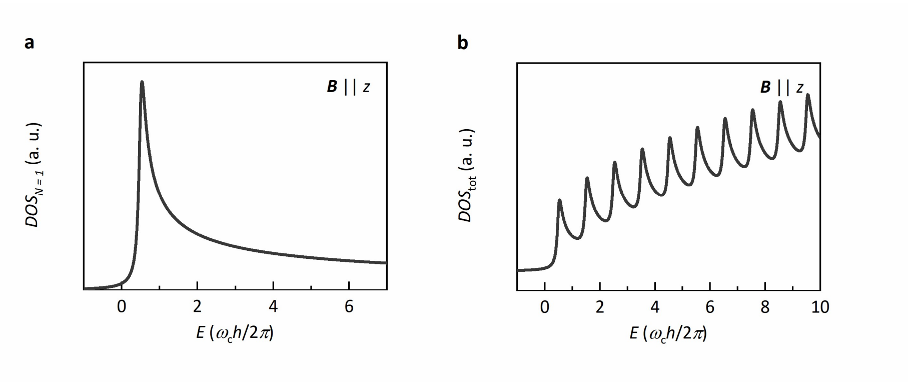

Supplementary Fig. S5. Density of states of Landau-level bands. a, Density of states $DOS_{N=1}$ of the $N = 1$ Landau level and **b**, total density of states $DOS_{\mathrm{tot}} = \sum_N DOS_N$ of a 3D Hall system as a function of energy E. DOS_N is the density of states of the Nth Landau level. To mimic the effects of disorder, we have included a Lorentzian level broadening for the Landau levels, which smoothens out the van Hove singularities at the band bottoms.

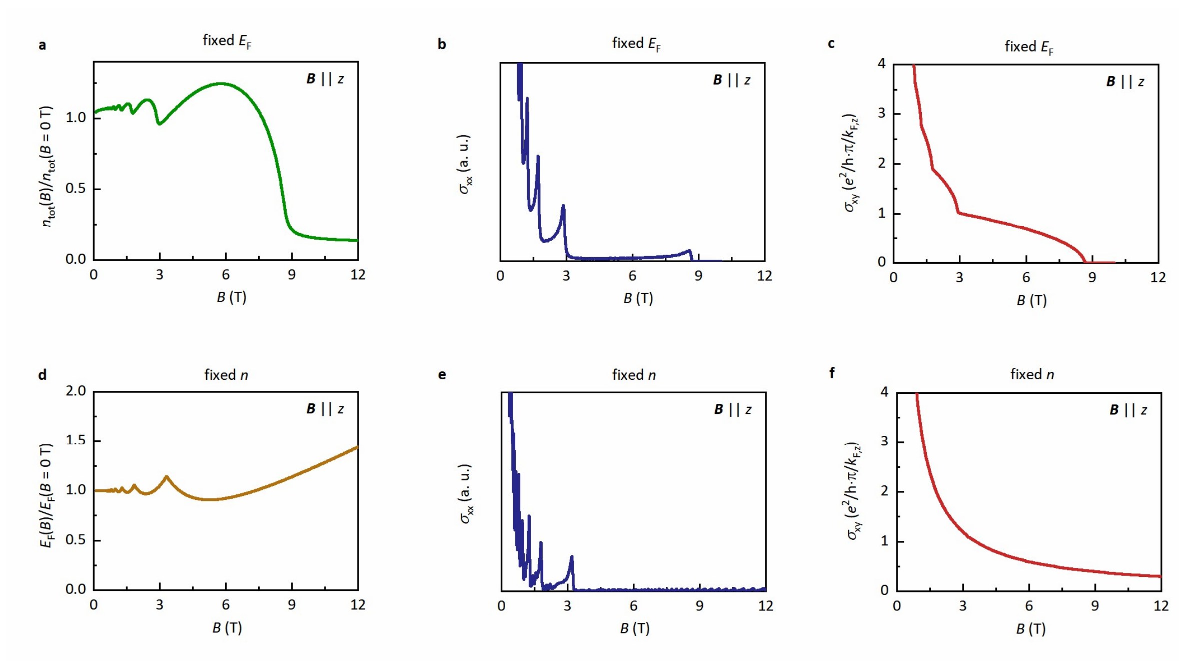

Supplementary Fig. S6. 3D Hall effect at fixed Fermi energy E_F and at fixed particle number n. a, Relative electron number, **b**, longitudinal conductivity σ_{xx} and **c**, Hall conductivity σ_{xy} normalized by the zero field Fermi wavevector k_{Fz} as a function of magnetic field $\boldsymbol{B}$ in z-direction for fixed E_F. In the calculations, we exemplarily choose the band mass $m = m^* = 0.067\ m_e$, where m_e, is the free-electron mass, the Fermi level 7.5 meV, and the Landau-level broadening 0.12 meV. **d**, Relative Fermi level, **e**, σ_{xx}, and **f**, σ_{xy} as a function of $\boldsymbol{B}$ in z-direction for fixed n. The same model parameters and zero-field electron density as for the calculations with fixed E_F were used, except for the level broadening and the transport lifetime, which were both divided by a factor of 10 in this case.

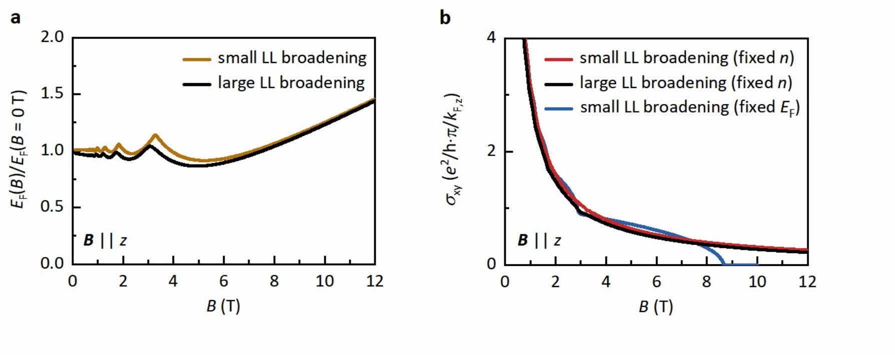

Supplementary Fig. *S*7. 3D Hall effect at fixed particle number for a larger level broadening. a, Relative Fermi level and **b**, Hall conductivity σ_{xy} normalized by the zero field Fermi wavevector k_{Fz} as a function of magnetic field $\boldsymbol{B}$ in z-direction. The yellow and red solid curves correspond to the same model parameters and zero-field electron density as in Supplementary Fig. S6d and f, respectively (small Landau level broadening and transport lifetime). The black curves use ten times larger level broadening and transport lifetime. For comparison, panel B also shows the Hall conductivity at fixed Fermi level from Supplementary Fig. S6c (blue curve).

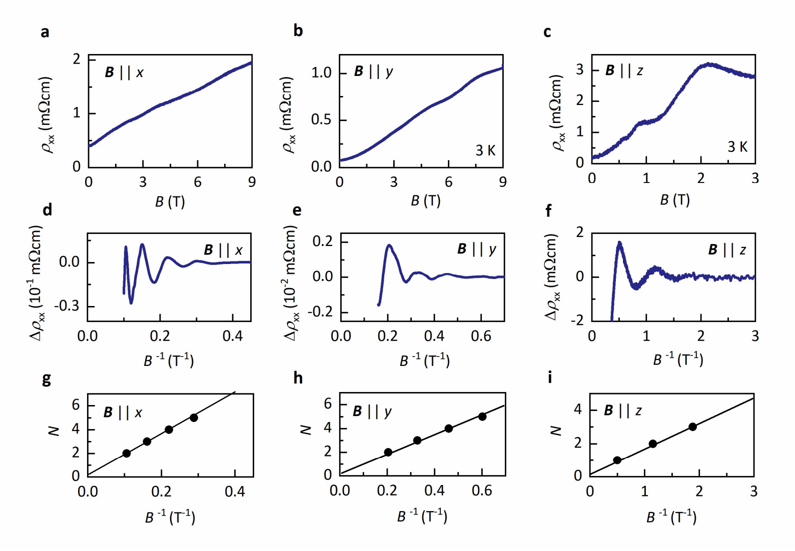

Supplementary Fig. S8. Analysis of the Shubnikov-de Haas oscillations of sample B at 3 K with the magnetic field applied along the three principal axes *x*, *y*, and *z*. **a**, ρ_{xx} as a function of ***B*** at 3 K with ***B*** applied along *x,* **b**, along *y* and **c**, along the *z*-direction. **d**, Variation of the longitudinal electrical resistivity $\Delta\rho_{xx}$ as a function of ***B*** at 3 K with ***B*** applied along *x,* **e**, along *y* and **f**, along the *z*-direction. **g**, Landau-index *N* fan diagram with ***B*** applied along *x,* **h**, along *y,* and **i**, along the *z*-direction.

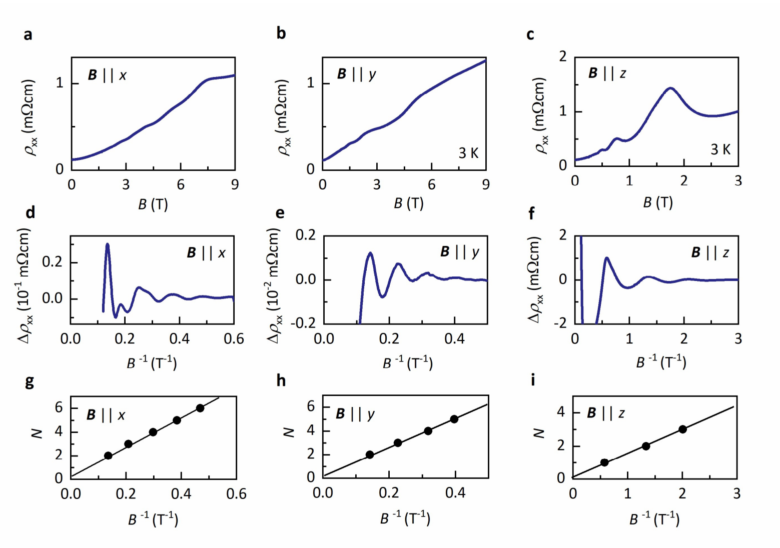

Supplementary Fig. S9. Analysis of the Shubnikov-de Haas oscillations of sample C at 3 K with the magnetic field applied along the three principal axes *x*, *y*, and *z*. **a**, ρ_{xx} as a function of $\boldsymbol{B}$ at 3 K with $\boldsymbol{B}$ applied along *x*, **b**, along *y* and **c**, along the *z*-direction. **d**, Variation of the longitudinal electrical resistivity $\Delta\rho_{xx}$ as a function of $\boldsymbol{B}$ at 3 K with $\boldsymbol{B}$ applied along *x*, **e**, along *y*, and **f**, along the *z*-direction. **g**, Landau-index *N* fan diagram with $\boldsymbol{B}$ applied along *x*, **h**, along *y*, and **i**, along the *z*-direction.

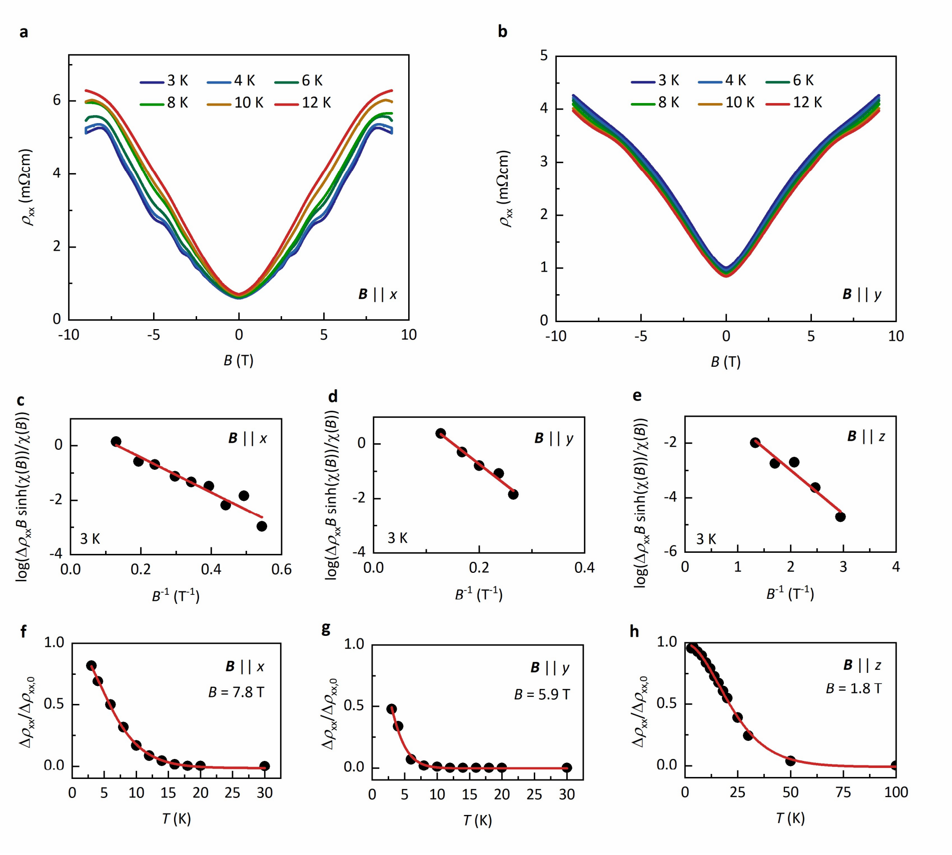

Supplementary Fig. S10. Determination of the mobility and cyclotron mass. a, ρ_{xx} as a function of $\boldsymbol{B}$ for various temperatures $T \geq 3$ K with $\boldsymbol{B}$ applied in x and **b**, in y. **c**, Dingle plots of $\Delta\rho_{xx}|\boldsymbol{B}|\sinh[\chi(T)]/\chi(B)$ versus $\boldsymbol{B}^{-1}$ with $\chi(B) = \frac{2\pi^2 k_B T m_c}{\hbar e \boldsymbol{B}}$ at 3 K with $\boldsymbol{B}$ applied along x, **d**, along y and **e**, along the z-direction. The lines are linear fits to the measurement data to obtain the electron mobility μ, as explained in Materials and Methods. **f**, The cyclotron masses m_c for $\boldsymbol{B}$ applied along x, **g**, along y and **h**, along the z-direction is obtained from fits (red lines) to $\Delta\rho_{xx}/\Delta\rho_{xx,0}$, where $\Delta\rho_{xx,0}$ is the extrapolated $\Delta\rho_{xx}$ at zero Kelvin. $\Delta\rho_{xx}$ for each principal direction is taken at the magnetic field of the lowest accessible Landau level.

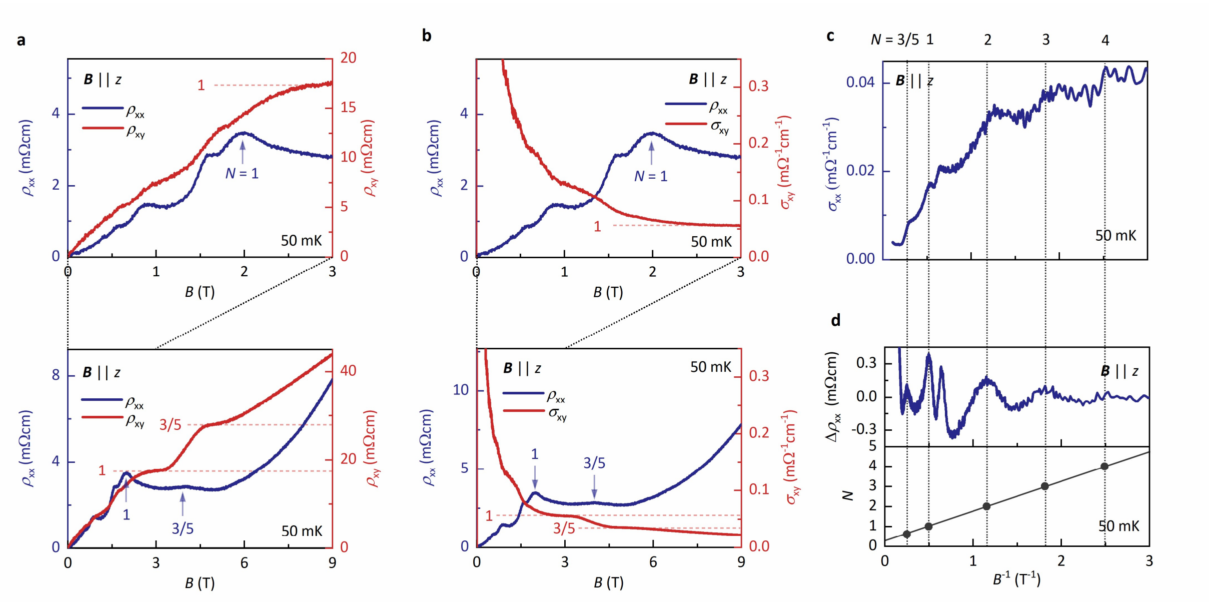

Supplementary Fig. S11. Low-temperature Hall response in $HfTe_5$ sample B. a, Longitudinal electrical resistivity ρ_{xx} (blue, left axis) and Hall resistivity ρ_{xy} (red, right axis) as a function of $\boldsymbol{B}$ at $T = 50$ mK with $\boldsymbol{B}$ applied in z for 0 T $\leq \boldsymbol{B} \leq$ 3 T (upper panel) and 0 T $\leq \boldsymbol{B} \leq$ 9 T (lower panel). The blue arrows mark the onset of a Landau level (LL). The blue numbers label the index N of the Landau level and the red numbers label the corresponding value of ρ_{xy} (or σ_{xy}) with respect to $(h/e^2)\pi/k_{F,z}$ (or $(e^2/h)k_{F,z}/\pi$, respectively). **b**, Longitudinal electrical resistivity ρ_{xx} (blue, left axis) and Hall conductivity σ_{xy} (red, right axis) as a function of $\boldsymbol{B}$ at T = 50 mK with $\boldsymbol{B}$ applied in z for 0 T $\leq \boldsymbol{B} \leq$ 3 T (upper panel) and 0 T $\leq \boldsymbol{B} \leq$ 9 T (lower panel). **c**, Longitudinal electrical conductivity σ_{xx} (blue, left axis) as a function of $\boldsymbol{B}^{-1}$ at T = 50 mK with $\boldsymbol{B}$ applied in z. **d**, Variation of the longitudinal electrical resistivity $\Delta\rho_{xx}$ as a function of $\boldsymbol{B}$ at 50 mK with $\boldsymbol{B}$ applied along z (upper panel) and Landau-index fan diagram (lower panel) as a function of $\boldsymbol{B}^{-1}$ at T = 50 mK with $\boldsymbol{B}$ applied in z.

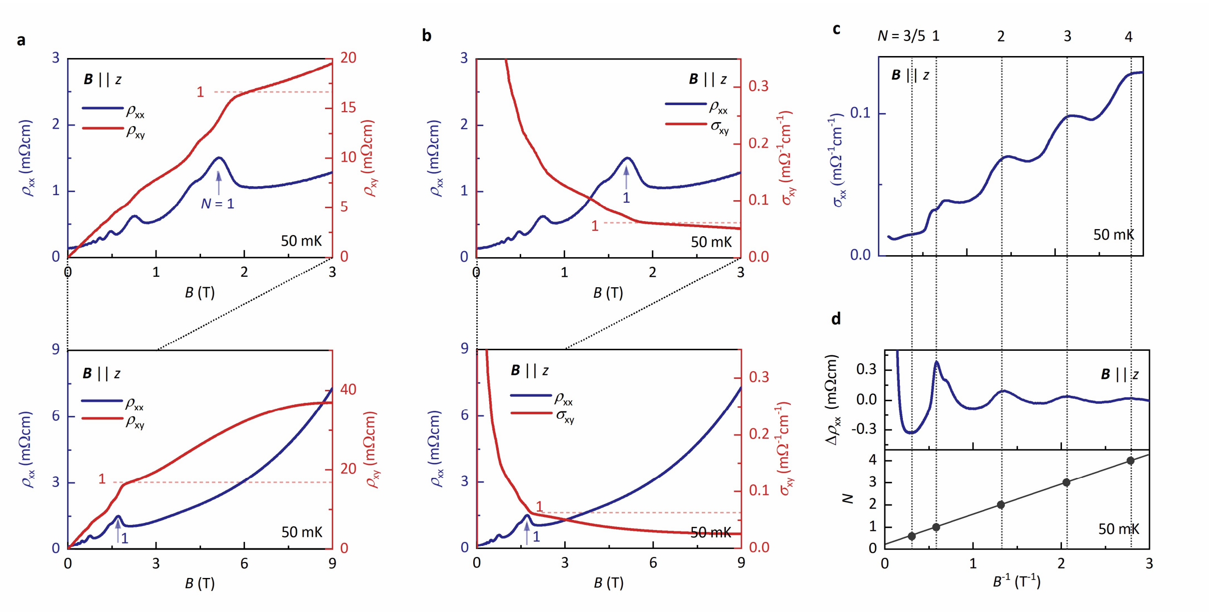

Supplementary Fig. S12. Low-temperature Hall response in $HfTe_5$ sample C. a, Longitudinal electrical resistivity ρ_{xx} (blue, left axis) and Hall resistivity ρ_{xy} (red, right axis) as a function of $\boldsymbol{B}$ at $T = 50$ mK with $\boldsymbol{B}$ applied in z for $0\ \mathrm{T} \leq \boldsymbol{B} \leq 3\ \mathrm{T}$ (upper panel) and $0\ \mathrm{T} \leq \boldsymbol{B} \leq 9\ \mathrm{T}$ (lower panel). The blue arrows mark the onset of a Landau level (LL). The blue numbers label the index N of the Landau level and the red numbers label the corresponding value of ρ_{xy} (or σ_{xy}) with respect to $(h/e^2)\pi/k_{F,z}$ (or $(e^2/h)k_{F,z}/\pi$, respectively). **b**, Longitudinal electrical resistivity ρ_{xx} (blue, left axis) and Hall conductivity σ_{xy} (red, right axis) as a function of $\boldsymbol{B}$ at $T = 50$ mK with $\boldsymbol{B}$ applied in z for $0\ \mathrm{T} \leq \boldsymbol{B} \leq 3\ \mathrm{T}$ (upper panel) and $0\ \mathrm{T} \leq \boldsymbol{B} \leq 9\ \mathrm{T}$ (lower panel). **c**, Longitudinal electrical conductivity σ_{xx} (blue, left axis) and Hall conductivity σ_{xy} (red, right axis) as a function of $\boldsymbol{B}^{-1}$ at $T = 50$ mK with $\boldsymbol{B}$ applied in z. **d**, Variation of the longitudinal electrical resistivity $\Delta\rho_{xx}$ as a function of $\boldsymbol{B}$ at 50 mK with $\boldsymbol{B}$ applied along z (upper panel) and Landau-index fan diagram (lower panel) as a function of $\boldsymbol{B}^{-1}$ at $T = 50$ mK with $\boldsymbol{B}$ applied in z.

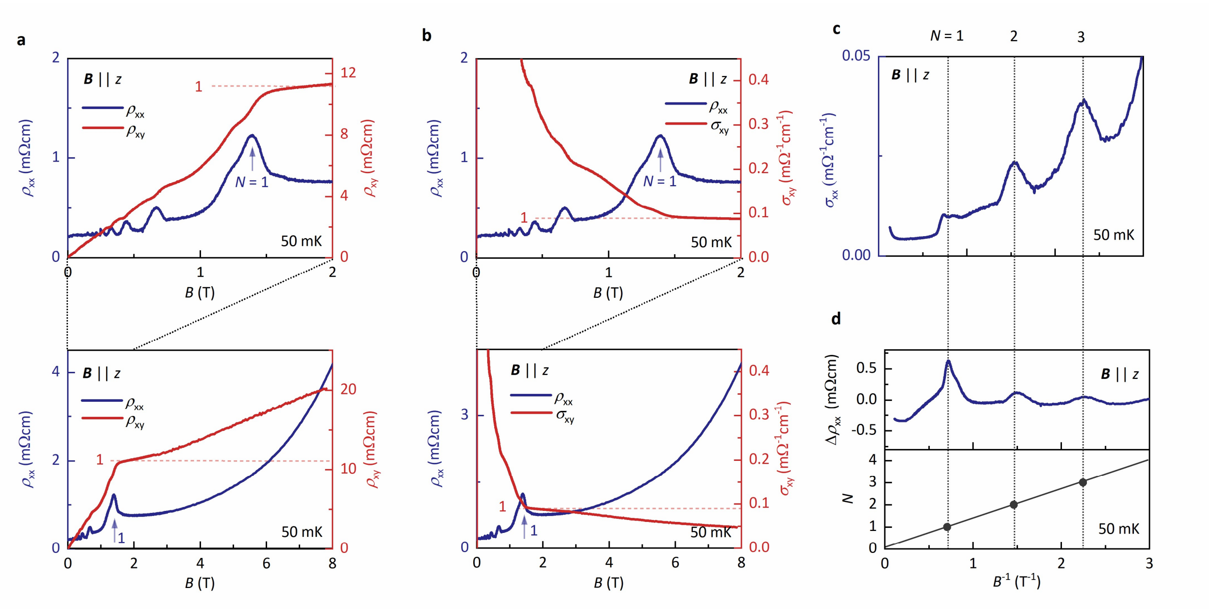

Supplementary Fig. S13. Low-temperature Hall response in $ZrTe_5$ sample D. a, Longitudinal electrical resistivity ρ_{xx} (blue, left axis) and Hall resistivity ρ_{xy} (red, right axis) as a function of $\boldsymbol{B}$ at T = 50 mK with $\boldsymbol{B}$ applied in z for 0 T $\leq \boldsymbol{B} \leq$ 3 T (upper panel) and 0 T $\leq \boldsymbol{B} \leq$ 9 T (lower panel). The blue arrows mark the onset of a Landau level (LL). The blue numbers label the index N of the Landau level and the red numbers label the corresponding value of ρ_{xy} (or σ_{xy}) with respect to $(h/e^2)\pi/k_{F,z}$ (or $(e^2/h)k_{F,z}/\pi$, respectively). **b**, Longitudinal electrical resistivity ρ_{xx} (blue, left axis) as a function of $\boldsymbol{B}$ at T = 50 mK with $\boldsymbol{B}$ applied in z for 0 T $\leq \boldsymbol{B} \leq$ 3 T (upper panel) and 0 T $\leq \boldsymbol{B} \leq$ 9 T (lower panel). **c**, Longitudinal electrical conductivity σ_{xx} (blue, left axis) and Hall conductivity σ_{xy} (red, right axis) as a function of $\boldsymbol{B}^{-1}$ at T = 50 mK with $\boldsymbol{B}$ applied in z. **d**, Variation of the longitudinal electrical resistivity $\Delta\rho_{xx}$ as a function of $\boldsymbol{B}$ at 50 mK with $\boldsymbol{B}$ applied along z (upper panel) and Landau-index fan diagram (lower panel) as a function of $\boldsymbol{B}^{-1}$ at T = 50 mK with $\boldsymbol{B}$ applied in z.

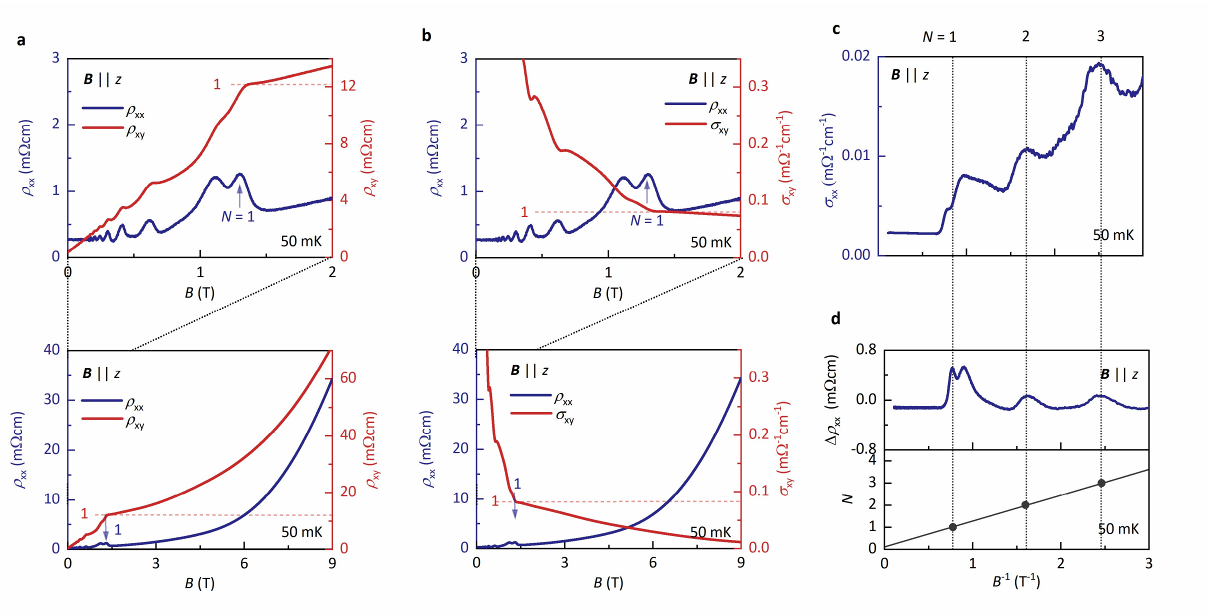

Supplementary Fig. S14. Low-temperature Hall response in $ZrTe_5$ sample E. a, Longitudinal electrical resistivity ρ_{xx} (blue, left axis) and Hall resistivity ρ_{xy} (red, right axis) as a function of $\boldsymbol{B}$ at $T = 50$ mK with $\boldsymbol{B}$ applied in z for $0\ \mathrm{T} \le \boldsymbol{B} \le 3$ T (upper panel) and $0\ \mathrm{T} \le \boldsymbol{B} \le 9$ T (lower panel). The blue arrows mark the onset of a Landau level (LL). The blue numbers label the index N of the Landau level and the red numbers label the corresponding value of ρ_{xy} (or σ_{xy}) with respect to $(h/e^2)\pi/k_{F,z}$ (or $(e^2/h)k_{F,z}/\pi$, respectively). **b**, Longitudinal electrical resistivity ρ_{xx} (blue, left axis) as a function of $\boldsymbol{B}$ at $T = 50$ mK with $\boldsymbol{B}$ applied in z for $0\ \mathrm{T} \le \boldsymbol{B} \le 3$ T (upper panel) and $0\ \mathrm{T} \le \boldsymbol{B} \le 9$ T (lower panel). **c**, Longitudinal electrical conductivity σ_{xx} (blue, left axis) and Hall conductivity σ_{xy} (red, right axis) as a function of $\boldsymbol{B}^{-1}$ at $T = 50$ mK with $\boldsymbol{B}$ applied in z. **d**, Variation of the longitudinal electrical resistivity $\Delta\rho_{xx}$ as a function of $\boldsymbol{B}$ at 50 mK with $\boldsymbol{B}$ applied along z (upper panel) and Landau-index fan diagram (lower panel) as a function of $\boldsymbol{B}^{-1}$ at $T = 50$ mK with $\boldsymbol{B}$ applied in z.

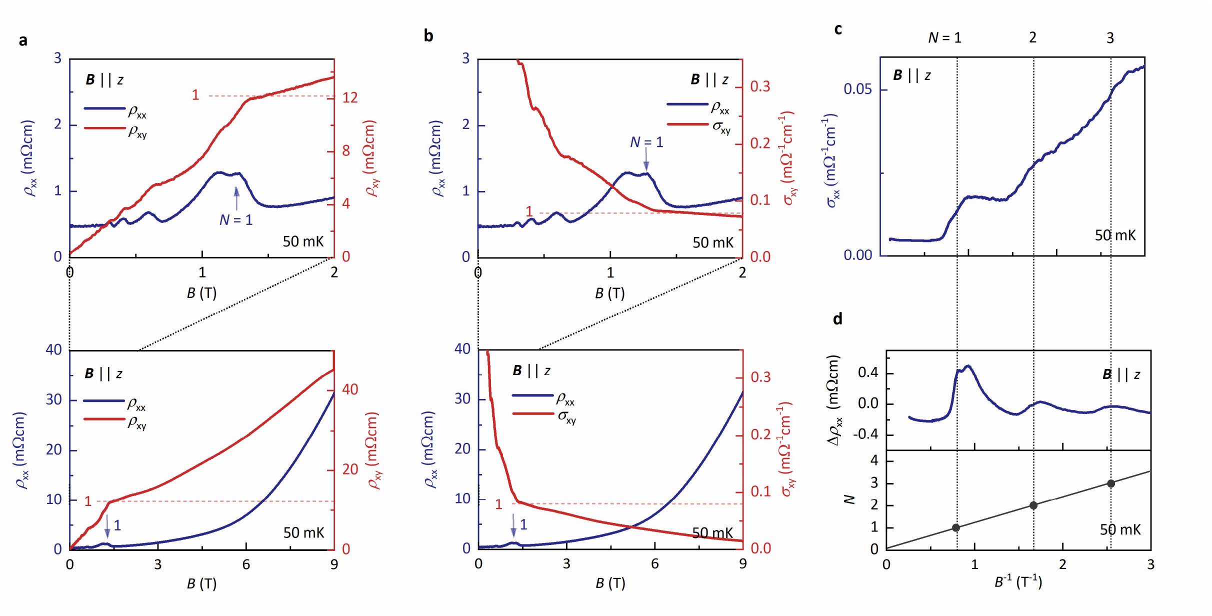

Supplementary Fig. S15. Low-temperature Hall response in $ZrTe_5$ sample F. a, Longitudinal electrical resistivity ρ_{xx} (blue, left axis) and Hall resistivity ρ_{xy} (red, right axis) as a function of $\boldsymbol{B}$ at $T = 50$ mK with $\boldsymbol{B}$ applied in z for 0 T $\leq \boldsymbol{B} \leq$ 3 T (upper panel) and 0 T $\leq \boldsymbol{B} \leq$ 9 T (lower panel). The blue arrows mark the onset of a Landau level (LL). The blue numbers label the index N of the Landau level and the red numbers label the corresponding value of ρ_{xy} (or σ_{xy}) with respect to $(h/e^2)\pi/k_{F,z}$ (or $(e^2/h)k_{F,z}/\pi$, respectively). **b**, Longitudinal electrical resistivity ρ_{xx} (blue, left axis) as a function of $\boldsymbol{B}$ at $T = 50$ mK with $\boldsymbol{B}$ applied in z for 0 T $\leq \boldsymbol{B} \leq$ 3 T (upper panel) and 0 T $\leq \boldsymbol{B} \leq$ 9 T (lower panel). **c**, Longitudinal electrical conductivity σ_{xx} (blue, left axis) and Hall conductivity σ_{xy} (red, right axis) as a function of $\boldsymbol{B}^{-1}$ at $T = 50$ mK with $\boldsymbol{B}$ applied in z. **d**, Variation of the longitudinal electrical resistivity $\Delta\rho_{xx}$ as a function of $\boldsymbol{B}$ at 50 mK with $\boldsymbol{B}$ applied along z (upper panel) and Landau-index fan diagram (lower panel) as a function of $\boldsymbol{B}^{-1}$ at $T = 50$ mK with $\boldsymbol{B}$ applied in z.

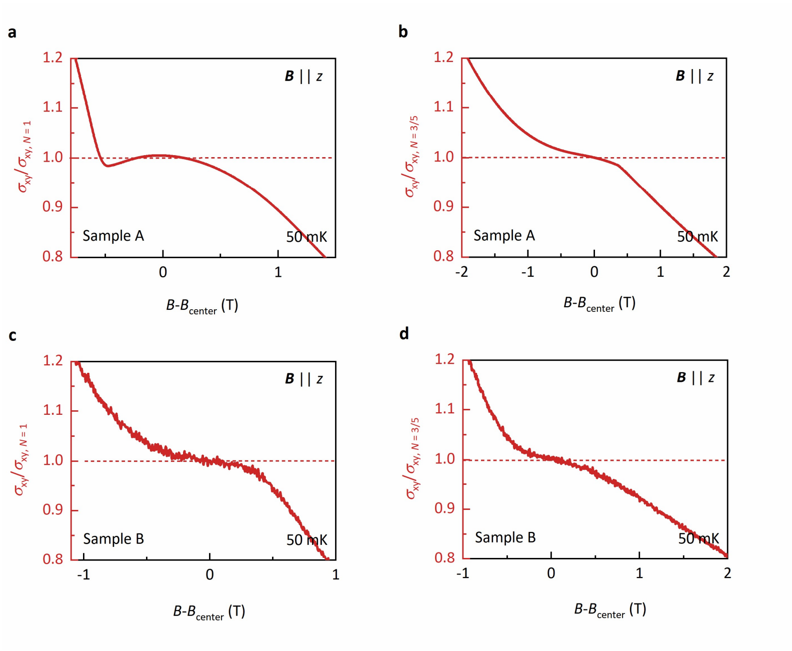

Supplementary Fig. S16. Flatness of the Hall plateaus of $HfTe_5$ sample A and B. a, Hall conductivity σ_{xy} relative to $\sigma_{xy,\,N=1} = 1 \cdot \frac{e^2}{h}\frac{k_{F,z}}{\pi}$ as a function of magnetic field B with respect to the center of the plateau B_{center} for Sample A at $T = 50$ mK with B applied in z. **b**, σ_{xy} relative to $\sigma_{xy,\,N=3/5} = 3/5 \cdot \frac{e^2}{h}\frac{k_{F,z}}{\pi}$ as a function of B with respect to B_{center} for Sample A at $T = 50$ mK with B applied in z. **c**, σ_{xy} relative to $\sigma_{xy,\,N=1}$ as a function of B with respect to B_{center} for Sample B at $T = 50$ mK with $\boldsymbol{B}$ applied in z. **d**, σ_{xy} relative to $\sigma_{xy,\,N=3/5}$ as a function of B with respect to B_{center} for Sample B at $T = 50$ mK with $\boldsymbol{B}$ applied in z.

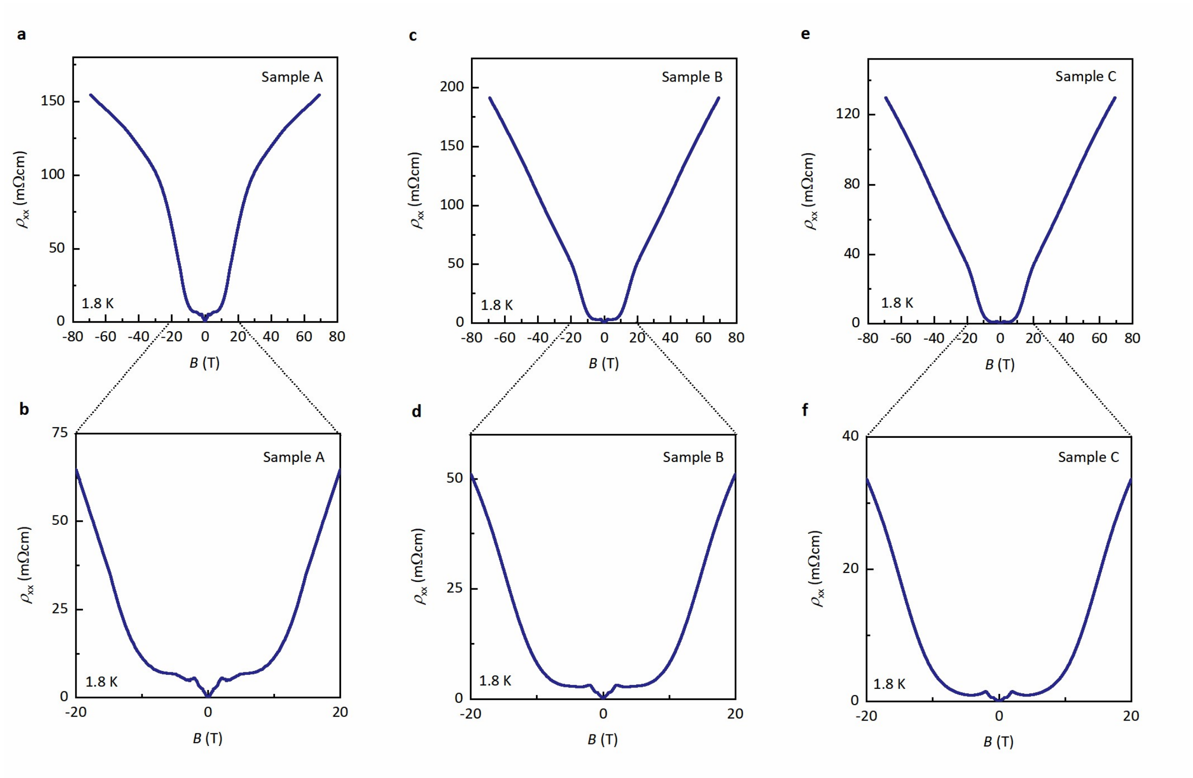

Supplementary Fig. S17. High-field magneto-transport. a, Longitudinal electrical resistivity ρ_{xx} of sample A at 1.8 K as a function of magnetic field $\boldsymbol{B} \parallel z$ up to ±70 T and **b**, up to ±20 T. **c**, ρ_{xx} of sample B at 1.8 K as a function of $\boldsymbol{B}$ up to ±70 T and **d**, up to ±20 T. **e**, ρ_{xx} of sample C at 1.8 K as a function of $\boldsymbol{B}$ up to ±70 T and **f**, up to ±20 T. For all samples investigated, no additional local maxima are observed above 9 T.

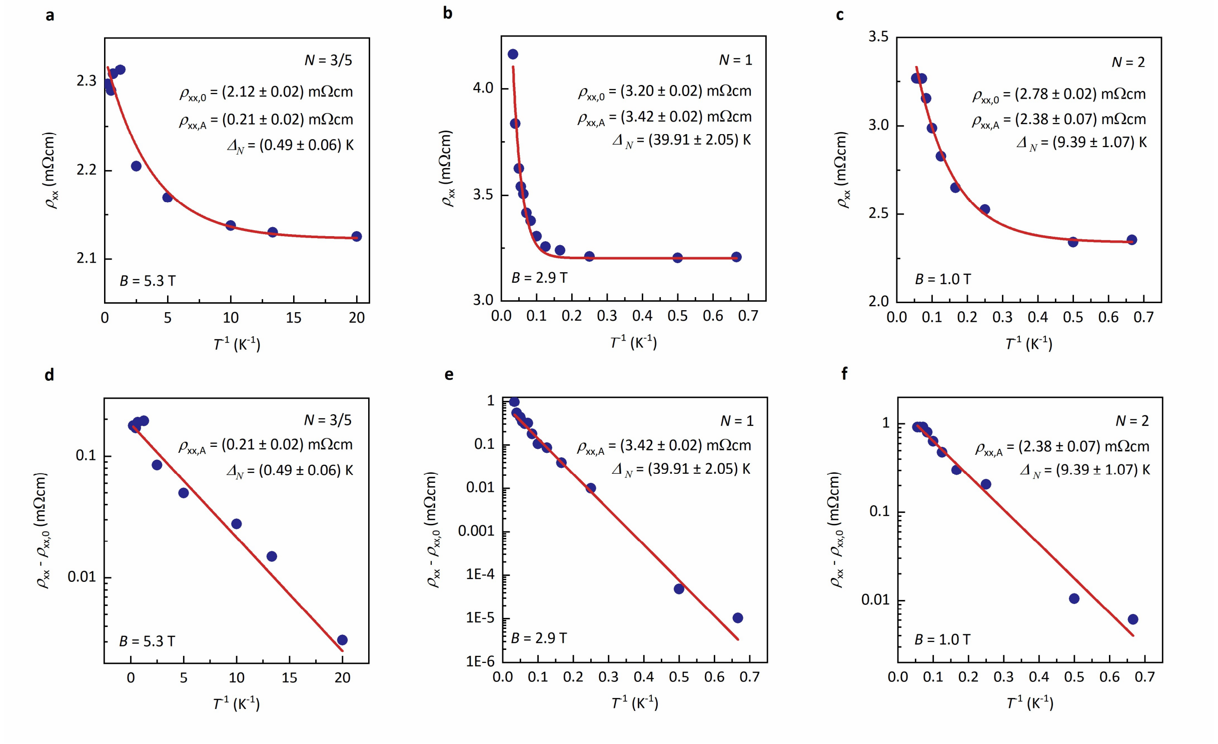

Supplementary Fig. S18. Fit of the gap energies. **a**, ρ_{xx} as a function of T^{-1} at the magnetic field B of the Shubnikov-de Haas minima of index $N = 3/5$, **b**, $N = 1$, **c**, $N = 2$. The gap energies Δ_ν are fitted (red lines) in the thermally activated regime $\rho_{xx}(B_z) = \rho_{xx,\mathrm{A}} \exp(-\Delta_N/2k_\mathrm{B}T) + \rho_{xx,0}$, where k_B is the Boltzmann constant, $\rho_{xx,\mathrm{A}}$ a scaling factor and $\rho_{xx,0}$ accounts for the finite ρ_{xx} in the Shubnikov-de Hass minima due to disorder broadening of the Landau levels. **d,** Log-plot of ρ_{xx} -$\rho_{xx,0}$ as a function of T^{-1} of the data and fits of a, **e**, b and **f**, c.

Supplementary Tables

Sample	Magnetic field's direction	SdH frequency B_F (T)	Fermi area S_F (10^{-4} Å^{-2})	Fermi wave vector k_F (10^{-3} Å^{-1})	Fermi wave length λ_F (nm)	Cyclotron mass m_c (m_0)	Effective mass m^* (m_0)	Fermi velocity v_F (10^5 m/s)	Dingle temperature T_D (K)	Lifetime τ (ps)
A	$\boldsymbol{B} \parallel x$	9.9 ± 0.1	9.9 ± 0.1	5.2 ± 0.9	120 ± 23	0.117 ± 0.002	0.034 ± 0.001	1.8 ± 0.5	0.17 ± 0.02	0.11 ± 0.01
	$\boldsymbol{B} \parallel y$	14.5 ± 0.2	14.5 ± 0.2	7.7 ± 1.5	82 ± 15	0.303 ± 0.019	0.005 ± 0.001	17.5 ± 3.5	0.07 ± 0.01	0.05 ± 0.01
	$\boldsymbol{B} \parallel z$	1.3 ± 0.1	1.3 ± 0.1	57.7 ± 5.9	11 ± 1	0.117 ± 0.002	2.714 ± 0.253	0.2 ± 0.1	0.69 ± 0.1	0.45 ± 0.06
B	$\boldsymbol{B} \parallel x$	8.4 ± 0.1	8.4 ± 0.1	5.3 ± 0.6	-	-	-	-	-	-
	$\boldsymbol{B} \parallel y$	12.9 ± 0.6	12.9 ± 0.6	8.2 ± 2.2	-	-	-	-	-	-
	$\boldsymbol{B} \parallel z$	1.4 ± 0.1	1.4 ± 0.1	48.3 ± 8.9	13 ± 2	-	-	-	-	-
C	$\boldsymbol{B} \parallel x$	8.8 ± 0.1	8.8 ± 0.1	5.9 ± 0.2	-	-	-	-	-	-
	$\boldsymbol{B} \parallel y$	10.5 ± 1.2	10.5 ± 1.2	7.0 ± 3.2	-	-	-	-	-	-
	$\boldsymbol{B} \parallel z$	1.3 ± 0.2	1.3 ± 0.2	47.6 ± 1.5	13 ± 2	-	-	-	-	-

Supplementary Table S1. Band-structure parameters of $HfTe_5$ sample A, B, and C, obtained from Shubnikov-de Haas oscillations. The variations denote the errors from the fits and from error propagation as explained in the methods and main text.

Supplementary References

1. Tang, F. *et al.* Three-dimensional quantum Hall effect and metal–insulator transition in ZrTe5. *Nature* **569**, 537–541 (2019).

2. Halperin, B. I. Possible states for a three-dimensional electron gas in a strong magnetic field. *Jpn. J. Appl. Phys.* **26**, 1913 (1987).

3. Endo, A., Hatano, N., Nakamura, H. & Shirasaki, R. Fundamental relation between longitudinal and transverse conductivities in the quantum Hall system. *J. Phys. Condens. Matter* **21**, 345803 (2009).

4. Mahan, G. D. *Many-particle physics*. (Springer Science & Business Media, 2013).
